# Return to flying after coronary artery disease: A case series among Malaysian pilots

**DOI:** 10.1002/1348-9585.12241

**Published:** 2021-06-21

**Authors:** Zulkefley Mohammad, Rosnah Ismail, Mohd Rafizi Mohamed Rus, Mohammed Haizar Haron

**Affiliations:** ^1^ Department of Community Health Faculty of Medicine University Kebangsaan Malaysia Cheras Malaysia; ^2^ Institute of Aviation Medicine, Subang Air Base Shah Alam Selangor Malaysia; ^3^ Cardiology Unit Department of Medicine Armed Forces Hospital Tuanku Mizan Kuala Lumpur Malaysia

**Keywords:** coronary artery disease, pilot, return to work, revascularization, risk assessment

## Abstract

**Objectives:**

Pilots with coronary artery disease (CAD) are at increased risk of myocardial infarction, stroke, and possibly death. Return to flying duties may be considered after a detailed risk assessment. The aim of this retrospective case series is to describe the return to flying duty process.

**Methods:**

We conducted a retrospective case review of pilots diagnosed with CAD at the Institute of Aviation Medicine (IAM), Royal Malaysian Air Force (RMAF) in October 2020.

**Results:**

Thirteen cases of CAD were included in the review. Ten pilots were diagnosed after developing acute coronary syndrome; the remaining three pilots were diagnosed during a routine medical examination via an exercise stress test. Twelve pilots required a revascularization procedure. A total of 11 pilots (84.6%) were recertified for flying duties, while another two were disqualified. The duration to recertification for these 11 pilots was between three months and one year.

**Conclusions:**

The risk assessment was initiated with initial risk‐stratification using population‐appropriate risk calculator combined with the 4 × 4 aeromedical risk matrix. The reassessment of return to flying after coronary artery disease must be carried out no sooner than six months after the event. Pilots must be hemodynamically stable with no evidence of significant inducible ischemic left and a minimum 50% of ventricular ejection fraction (LVEF). A follow‐up is recommended at the initial six months after recertification and then annually with a routine noninvasive cardiac assessment.

## 1 INTRODUCTION

Pilots are required to be fit and healthy since they experience constant levels of high physical and mental stress in the scope of their daily work. They undergo periodic medical evaluations to ensure physiological, cognitive, and emotional readiness. Pilots with risk factors for cardiovascular disease (CVD) such as hypertension or diabetes typically are motivated to control the underlying medical conditions in order to retain their flight career.^1^ Pilots are constantly exposed to operational stressors as well as environmental stressors. Common operational stressors include extended flights, shift work, irregular mealtimes, stress, fatigue, and biorhythm disturbances with changing time zones. On the other hand, environmental stressors such as hypoxia and hypobaric conditions caused by high‐altitude conditions may strain the cardiorespiratory system.^2^ Compared to civilian pilots, military pilots have an additional burden of stress due to exposure to the acceleration of gravity forces while performing aerobatics or air combat maneuvers.^2^ Both operational and environmental stressors increase the risk of strain to the cardiorespiratory system and lead to the establishment of CAD.

Pilots that show evidence of CAD, either incidentally discovered during health screening or presenting with cardiac symptoms (eg chest pain or breathlessness), must undergo serial reassessment and recertification to return to their flying duties. According to the Civil Aviation Authority of Malaysia (CAAM) guidelines, the reassessment and recertification for a return to flying duties for a civilian or private pilot can be completed six months after the cardiovascular event.^3^ For a military pilot, however, a one year waiting period is required.^4^ Thus, the aim of this paper is to describe the return to flying process for a pilot with CAD. The aim of this report is to provide a guide for occupational health practitioners to medically certify a pilot for return to flying when requested by an individual of the relevant industry.

## 2 METHOD

A retrospective case review was conducted in October 2020 at the Institute of Aviation Medicine (IAM), Royal Malaysian Air Force (RMAF). Medical records of pilots with CAD was retrieved from the list of the Aircrew Medical Panel of RMAF and the Review Medical Board of CAAM. Only participating pilots diagnosed with plaque lesions in the coronary artery were included, while pilots with a primary diagnosis of arrhythmia were excluded. Participants with pending recertification or incomplete information were excluded. The selected participants were contacted and provided with all information on the study. Once they agreed, the written consent was taken. The anonymity of all the participating pilots was guaranteed by the creation of case numbers.

The risk factors of all the cases were stratified using the general cardiovascular risk profile in the primary care setting, known as Framingham Risk Score (FRS) 2008.^5^ The risk stratification estimates the 10‐year risk of presenting with clinical CVD, including CAD, stroke, peripheral vascular disease (PVD), and cardiac death. The variables included in the risk stratification were age, gender, total cholesterol, HDL cholesterol, systolic blood pressure, medication for hypertension, smoking status, presence of diabetes, and history of known CVD (CAD, PVD, or stroke). Pilots with a 10‐year risk of a CVD event less than 10% were categorized as low risk, while those with a 10‐year risk of a CVD event between 10% and 20% were in a moderate risk group. Pilots with a CVD risk greater than 20% were categorized as high risk.

## 3 RESULT

Thirteen completed cases were retrieved from the list of the Aircrew Medical Panel of RMAF and the Review Medical Board of CAAM. All pilots were male, and the youngest was 35 years old while the oldest was 58 years old. The majority of the pilots [N = 7, 53.8%] were government pilots (military and police), and six were doing administrative work (nonactive flyers). All civilian pilots (commercial and private) were active flyers. Table 1 shows the characteristics of pilots with CAD. Although the pilot in case six was not an active flyer, he was given the flying limitation ‘as or with a co‐pilot’ prior to the diagnosis of CAD. This limitation was imposed due to his 10‐year risk of a CVD event being greater than 20%. Surprisingly, the other four cases (Cases 4, 10, 11, and 13) had no flying restrictions although their 10‐year risk of a CVD event was greater than 20%.

**TABLE 1:** Characteristic of the pilot with the CAD

Case No.	Age (year)	Type of Aircraft and total flying hours (H)	Operational status prior to the cardiac event	Risk factors	The 10‐year risk of CVD event	Way of diagnosis	Angiogram findings	Cardiac intervention	Time for initial review	Postintervention parameters	Flying status
1	44	Military‐Fix wing fighter (1027H)	Desk Job	Family history Dyslipidaemia	9.4%	Positive EST	20% occlusion at LAD	Drug therapy	3 months		Fit
2	46	Private‐Helicopter (3629H)	Active flyer	Hypertension Overweight Dyslipidaemia	18.4%	Positive EST	80% occlusion at RCA	PCI to RCA	3 months	Perfusion scan normal, LVEF >55%	Fit, multicrew
3	43	Military‐Fix wing transport (4562H)	Desk Job	Smoker Dyslipidaemia	15.6%	Acute MI	90% occlusion at LAD, 45% LCX	PCI to LAD	1 year	Evidence of RWMA, LVEF<40%	DQ
4	46	Military‐Fix wing fighter (1235H)	Desk job	Hypertension Diabetes Dyslipidaemia	25.3%	Acute MI	Severe 2VD with tight left main stem	CABG	1 year	Evidence of RWMA, LVEF<40%	DQ
5	35	Military‐Rotary wing (1165H)	Desk job	Smoker Overweight	11.2%	Acute MI	90% occlusion at LCX	PCI to LCX	1 year	EST normal, normal, LVEF >55%, mild LVH	Fit, multicrew
6	56	Military ‐Fix wing transport (3670H)	Desk job	Hypertension	25.3%	Acute MI	90% occlusion at RCA	PCI to RCA	1 year	EST normal, normal, LVEF >55%	Fit, multicrew
7	59	Police‐Helicopter (4927H)	Desk job	Dyslipidaemia	18.4%	Angina	90% occlusion at LAD	PCI to LAD	1 year	EST normal, normal, LVEF >55%	Fit, multicrew
8	43	Police‐Helicopter (2854H)	Active flyer	Family history	6.7%	Acute MI	90% occlusion at RCA, 80% at LCX	PCI to RCA and LCX	6 months	Perfusion scan normal, LVEF >55%	Fit, multicrew
9	51	Private‐Helicopter (4480H)	Active flyer	Hypertension	18.4%	Positive EST	80% occlusion at LAD	PCI to LAD	6 months	Perfusion scan normal, LVEF >55%	Fit, multicrew
10	54	Private‐Helicopter (5075H)	Active flyer	Hypertension Diabetes Obesity	25.3%	Acute MI	80% occlusion at LAD	PCI to LAD	1 year	Perfusion scan normal, LVEF >55%	Fit, multicrew
11	58	Private‐Helicopter (5780H)	Active flyer	Hypertension Diabetes Obesity	30%	Acute MI	90% occlusion at LAD, 45% at LCX	PCI to LAD	1 year	Perfusion scan normal, LVEF >55%	Fit, multicrew
12	45	Commercial ‐Fix wing transport (5835H)	Active flyer	Nil	5.6%	Acute MI	80% occlusion at RCA	PCI to RCA	6 months	Perfusion scan normal, LVEF >55%	Fit, multicrew
13	56	Commercial ‐Fix wing transport (69040H)	Active flyer	Overweight Dyslipidaemia	21.6%	Acute MI	90% occlusion at LAD	PCI to LAD	6 months	Perfusion scan normal, LVEF >55%	Fit, multicrew

Abbreviations: EST, exercise stress test; DQ, disqualified; LAD, left anterior descending artery; LCX, left circumflex artery; LVEF, left ventricular ejection fraction; LVH, left ventricular hypertrophy; MI, myocardial infarction; PCI, percutaneous coronary intervention; RCA, Right coronary artery.

Three pilots were asymptomatic and were diagnosed with CAD after a positive exercise stress test (EST) while the remaining 10 pilots developed symptoms of an acute coronary syndrome (ACS). Twelve pilots were undergoing revascularization (eleven PCI and one CABG), including two pilots with no symptoms of ACS. Eleven pilots (84.6%) were recertificated for flying duties, while two were disqualified. The duration to recertification of these 11 pilots was between three months to one year. Of the 12 pilots who underwent revascularization, only one pilot (Case 2) was reassessed and recertificated after three months of revascularization, while the others were reassessed after six months of revascularization. Two military pilots (Cases 3 and 4) were disqualified from returning to flying duties since echocardiograms showed global hypokinesia and LVEF less than 50% even after 4 years of the recertification process. The duration to recertification for cases 11 and 12 were delayed for one year since the risk factors (hypertension and diabetes) were not yet controlled.

Cases 1 and 2 are explained in detail as follows.

### 3.1 Case 1 – Asymptomatic CAD without revascularization

The pilot, a 44‐year‐old fighter pilot (nonactive flyer with administrative duties), was a nonsmoker and had a strong family history of CAD. He had normotensive blood pressure (BP = 132/88 mmhg) with a BMI of 25.4 kg/m^2^. He was noted to have hypercholesterolemia. *Labs*: Total/HDL‐C = 5.8/0.85 mmol/L, LDL 5.0, Tg 2.1, FBS 4.9 mmol/L *Framingham Risk Score 2008*:9.4% of estimated 10‐year global CVD risk (low risk). He had abnormal ECG findings during a routine medical check‐up; a subsequent exercise stress test (EST) showed a positive result (asymptomatic). He was then subjected to coronary angiography which showed 20% stenosis of mid‐left anterior descending (LAD). His echocardiography showed no abnormality with a left ventricular ejection fraction (LVEF) of 70%. *Intervention*: Dietary modification, exercise program, and statin treatment. After 3 months, blood pressure was 118/72 and BMI was 23.8 kg/m^2^. *Labs*: Total/HDL‐C = 4.8/1.1 mmol/L, LDL 3.8, Tg 1.5, FBS 4.8 mmol/L. He was given fitness to fly without limitation with yearly cardiology review.

### 3.2 Case 2 – Asymptomatic CAD with revascularization

The pilot, a 46‐year‐old ex‐military pilot, was working as a helicopter pilot at a private company. He was a nonsmoker, with no family history of heart problems. He had been diagnosed with hypertension for three years and prescribed Amlodipine 10 mg daily. Blood pressure was 142/88 mmHg, and BMI was 27.2 kg/m^2^. *Labs*: Total/HDL‐C = 5.7/0.8 mmol/L, LDL 4.7, Tg 3.9, FBS 4.6 mmol/L *Framingham Risk Score 2008*:18.4% of estimated 10‐year global CVD risk (moderate risk). During his annual medical check‐up, he had abnormal ECG findings. He was subsequently subjected to an exercise stress test, and the result was positive. An echocardiogram at that time showed no abnormity with a left ventricular ejection fraction (LVEF) of 76%. His interventricular septal end diastole and end systole (IVsd) was 1.2 cm and all heart valves were normal. Subsequently, he underwent a coronary artery CT, and the outcome showed an Agatston calcium score of 170 with chronic total occlusion (CTO) at the mid‐right coronary artery (RCA) and collateralized by small antegrade and retrograde filling. The proximal LAD has 20% blockage, meanwhile LCX recessive was normal. *Intervention*: A percutaneous coronary intervention (PCI) to RCA was done with SYNERGY 4.0 mm coronary stent (post dilated using a 4.5 mm NC balloon), which was uneventful. *Medication*: Clopidogrel 75 mg daily, Aspirin 100 mg daily, Fenofibrate 145 mg daily, Rosuvastatin 10 mg daily, Amlodipine 10 mf daily, and Pantoprazole 40 mg daily. He returned after three months and was asymptomatic with NYHA Class I. He underwent a second EST, which was negative. A myocardial perfusion scan with Technetium‐99m tetrofosmin showed there was no significant inducible ischemic with LVEF >60%. His blood pressure (122/80 mmHg), cholesterol level, and blood sugar level were within the normal range. He was given the recertification fitness to fly with the limitation of multicrew operation as or with a co‐pilot and to be reviewed again after six months.

## 4 DISCUSSION

This article aims to describe the return to flying assessment for a pilot with CAD with the purpose of educating occupational health practitioners and other professionals with relevant expertise. Our report may provide a template to medically certify a pilot for return to flying. We highlighted two cases of asymptomatic CAD (with and without revascularization) because these are the possible scenario that may encounter by the occupational health practitioner during a routine medical examination for the pilot.

Fitness to fly for pilots is determined by an agreed threshold between acceptable and unacceptable medical incapacitation (eg heart attack or stroke) during flying. The threshold is referred to as the 1% rule, that is, a 1% per annum risk of medical incapacitation.^6^ In aviation medicine, the human ‘system’ has an acceptable failure risk, in the same way that an engineer determines a suitable threshold for the failure of other aircraft systems. The risk threshold for an acceptable level of controlled risk of acute incapacitation in a dual pilot operation is 1% per annum. This percentage is derived using engineering concepts to ensure the incidence of a fatal aircraft accident due to any pilot subsystem (ie, 1/100 of the overall 1 per 10^7^ hours of flying risk) is no greater than 1 per 10^9^ flying hours. In other words, the 1% rule is a risk threshold between acceptable and unacceptable risk. If the risk of medical incapacitation from CAD or any side effect of CVS medication is greater than 1% during the year, the pilot will be denied a medical certificate. However, the 1% rule is significantly limited since it was based on a series of assumptions relevant to 1‐hour commercial flights with critical flight times limited to take‐off and landing (6 min). Cardiovascular events or any other medical events are assumed to result in complete incapacitation of one pilot; a co‐pilot is assumed to be able to safely deal with a disability of the other pilot occurring during a critical period of landing and take‐off, 99 times out of 100. Therefore, a 2% risk per year (or up to 5% per year in certain circumstances) has been determined to be acceptable. Consequently, following the 1% rule, the pilot in Case 2 will be denied for recertification as the annual event rates of restenosis is 2%‐4% per year.^7^

Risk assessment is begun by performing an initial risk‐stratification using a population‐appropriate risk calculator. The risk assessment classifies risk as low (<10%/decade or <1%/year), intermediate (10%–20%/decade or 1%–2%/year), and high (>20%/decade or >2%/year).^6^ In this study, the risk‐stratification was based on the FRS 2008 calculator that used the lipid profile‐based formula. The FRS yielded a higher estimation of 10‐year CVD risk [median (IQR) = 18.4% (15.0%)] compared with the study performed in the general population of Malaysia [median (IQR) = 13.2% (14.0%)].^8^ Based on the FRS alone, all cases (except Case 1) should be grounded temporarily for further investigation since their FRS was greater than 10%.

**FIGURE 1:**
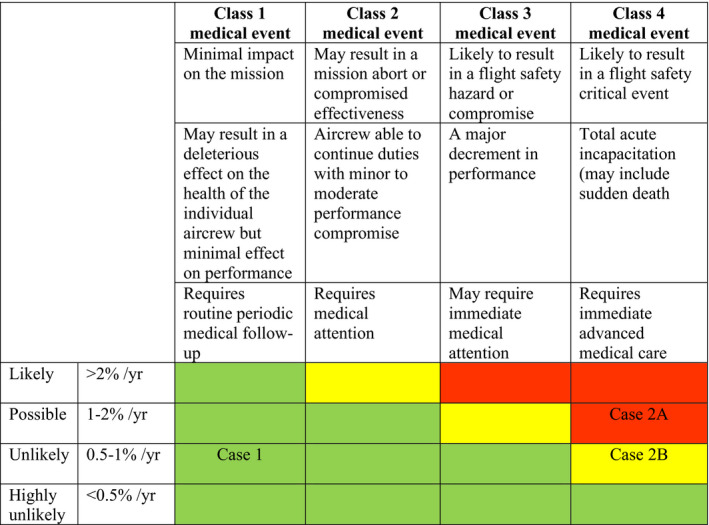
The 4 × 4 aeromedical risk matrix for a pilot (Adopted from Gray et al. 2018).^6^ *Note*. Case 2A referred to the aeromedical risk prior revascularisation, meanwhile Case 2B referred to aeromedical risk at three months post revascularisation.

The initial risk assessment can be carried out using the 4 × 4 aeromedical risk matrix, as shown in Figure 1. The risk matrix incorporates the likelihood and class of medical events. The classification of medical events, with the potential impact, and recommended medical interventions was taken from the Royal Canadian Air Force (RCAF).^6^ The green colour indicates acceptable risk, the yellow indicates tolerable with consent or limitation and red is an intolerable risk. In Case 1, the FRS was low (9.4%) [the likelihood of an event is unlikely (<1% per year)], and the probability of a medical event that can jeopardize flight safety was minimal since he had a minimal CAD. Thus, he was given full recertification with yearly cardiology assessment. The pilot in Case 2 had a FRS of 18.4% (likelihood is possible); a thorough investigation showed that he was categorized into a Class 4 medical event. Thus, he was temporarily grounded until he completed advanced medical care (marked as Case 2A in Figure 1). After the cardiac intervention, his FRS was 9.4%. This number indicates that he has low risk (unlikely). However, he is still at risk for late in‐stent thrombosis or the possibility of a major cardiovascular event (MACE).^8^ Therefore, he was given a flying limitation as a dual pilot operation (marked as Case 2B in Figure 1). As noted earlier, four pilots (Cases 4, 10, 11, and 13) did not have flying restrictions prior to their CAD diagnosis. The possible explanation for this is that their EST was negative. However, based on the 4 × 4 aeromedical risk matrix, they fall under the ‘yellow zone’ since they have 10‐year risk of a CVD event with a Class 2 medical event that is greater than 20%. These pilots should be restricted to dual pilot operations. The pilot in Case 4 should not be allowed to fly fighter aircraft. Although some of these pilots were not active flyers, these restrictions can provide important motivation for them to improve their health.

According to standard practices in the Malaysian military, individuals that have a CVD risk greater than 10% over 10 years or are over 40 years of age must have an exercise stress test (EST) since it is cost‐effective and readily available. However, previous studies have showed that the positive predictive value for EST is low in populations with a low prevalence of CAD, such as in military or pilot population. This may explain why military pilots such as the pilots in Cases 3, 4, 5, and 6 developed MI although they have been screened with EST. The pilot in Case 5 underwent EST prior to the event as it was a requirement before he attended an international course in Europe. Unfortunately, he developed MI during the course. Thus, according to the latest study, a pilot with an increased risk of a cardiovascular event should undergo enhanced screening with a coronary artery calcium score (CACS) or in combination with a CT coronary angiogram.^9^

If the lesion at LAD is increased to 30%‐49% or 30%‐40% at LMS or proximal LAD, the pilot should be given a flight restriction (nonhigh‐performance aircraft) as recommended by Davenport E. D. et al^10^ A pilot with any stenosis between 50% and 70% should undergo fractional flow reserve (FFR) assessment to verify hemodynamic significance. Pilots with FFR values of greater than 0.8 with more than one stenosis >50% should be disqualified or temporarily grounded from flying duties. In addition, they need to be aggressively treated according to the clinical guidelines. Pilots with FFR values less than 0.8 may be allowed restricted flight duties (nonperformance aircraft and multicrew‐operation).^7^, ^10^

Although revascularization looks promising for recertification of flying fitness, a pilot who undergoes a revascularization procedure are critically assessed for the following three elements: (1) type of revascularization (PCI vs CABG); (2) expected reoccurrence rates in the areas of revascularization; and (3) residual disease burden (including assessment of LVEF and regional wall motion, scar burden, and viability). This requires a multidiscipline approach between the cardiologist, cardiovascular surgeon, and aviation medical examiner (AME). The pilot in Case 4 underwent CABG for severe two‐vessel diseases and left main (LM) CAD as indicated by the clinical guidelines. The choice of intervention must be based on existing evidence. A study by Head SJ et al showed that all‐cause mortality was significantly higher with PCI compared with CABG.^11^ However, a subanalysis of the study showed that in nondiabetic patients with multivessel disease, PCI was as safe and effective as CABG. For a diabetic patient, CABG has a better outcome compared with PCI. In contrast, for revascularization using PCI, most of the bare‐metal stents and drug‐eluting stents (DES) are acceptable for pilots, with the exception of nonstent (plain old balloon) angioplasty (POBA), which has early restenosis rates as high as 35% which most often occurs within the first two to three months.^7^ For case 2, a SYNERGY stent was used for the PCI. It is a metal stent with a special drug coating added to help reduce the chance of the artery becoming blocked again.

Relatively hypoxic environments with oxygen concentrations around 15.2%‐17.6% in normal cabin pressure (5000 to 6000 feet) may compromise the cardiorespiratory system of pilots with CAD. Thus, it is crucial to ensure that these pilots show no evidence of significant inducible ischemic or regional wall motion abnormalities (RWMA) and have at least 50% of LVEF before allowing flight duties. This review showed that all military pilots were reassessed after one year of a revascularization procedure in accordance with military guidelines using EST and echocardiography or stress‐echocardiography. The guideline did not specify the modality used for assessing the residual disease burden. However, the best modality is noninvasive functional imaging, such as a myocardial perfusion scan.^3^, ^12^ Neither EST nor revascularization are highly recommended as a sole investigative modality for assessing pilots post‐MI since these techniques have low sensitivity to determine ischemia compared to noninvasive functional imaging.^13^

Restricted return to flying duties (nonperformance aircraft and multicrew operation) is possible after PCI or CABG. The Federal Aviation Administration (FAA) of the United States Department of Transportation has stated that pilots with uncomplicated MI, or who undergo PCI that excludes the left main coronary artery and CABG, can be recertificated as early as three months after the cardiovascular event.^14^ The recertification process for the pilot in Case 2 was similar to the FAA guidelines as the pilot did not have MI and only had PCI to RCA. In addition, his modifiable risk factors were under control. However, it is generally recommended to wait for six months due to the risk of restenosis, late in‐stent thrombosis, or the possibility of MACE.^7^

In evaluating return‐to‐work of pilots with CAD the cardiopulmonary exercise (CPX) test was not performed as all the pilots have good effect tolerance (except cases 3 and 4). All of them were asymptomatic and had EF of more than 50%. For cases 3 and 4, since their effort tolerance was reduced with significantly lower EF, they were straightly grounded from flying duties. CPX test is among the best prognosticators for medically managed advanced heart failure. For cases 3 and 4, the CPX test is helpful to evaluate their exercise capacity and predict the outcome of heart failure. Subsequently, the treatment can be optimized for a better outcome.

**FIGURE 2:**
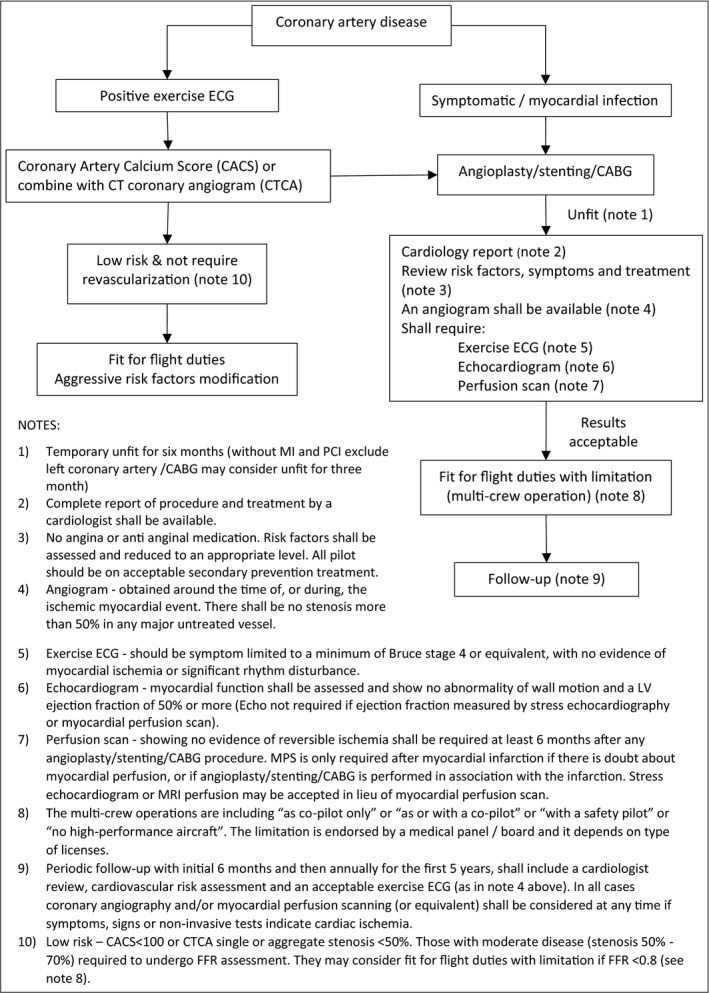
Flow chart for aeromedical disposition and recommendations for coronary artery disease (Adapted from UK CAA).^12^

After returning to flight, it is highly recommended that pilots regularly follow‐up with their primary care practitioners, AMEs, and cardiologists.^7^ It is important to assess the cardiac symptoms and vital signs (such as blood pressure and heart rate) and to ensure that each pilot has adopted lifestyle modifications, including abstinence of tobacco and compliance with medication. In Malaysia, the standard first follow‐up after recertification of flying duty is six months with the AME, since they are given medical licenses for an initial six months. A cardiologist report may be needed if they have symptoms such as chest pain or reduced effort tolerance. The next follow‐up is in six months (one year after revascularization) with a report and noninvasive cardiac assessment. Figure 2 shows a flow chart for aeromedical disposition and recommendations for CAD.

In this review, those who were active flyers have more flying hours. It may suggest that operational and environmental stressors may contribute to the development of CAD. However, at the same time, most of them have a co‐morbidity. Thus, further study needs to carry out to determine the factors associated with CAD among active flying pilots. There may be some limitation to this study since risk stratification using the FRS 2008 calculator may not reflect the true risk of the pilot. In addition, all military pilots who developed CAD were not actively flying since they had administrative jobs. From the records, we could not find any active military pilot with CAD. A possible explanation may be that active military flyers are much younger and healthier, or administrative jobs are stressful, which causes the non‐active military pilot to develop CAD. Nevertheless, this study is beneficial by highlighting the necessity to follow the guidelines provided to ensure flight safety, especially for the AME. In addition, the respective aviation authorities in Malaysia should update the aviation medicine guidelines pertaining to CAD based on the latest scientific evidence.

## 5 CONCLUSION

Pilots with CAD have an increased risk of myocardial infarction, stroke, and possibly death. As a result, pilots with CVD risk that is greater than intermediate (>10%/decade or >/year) should be investigated thoroughly. However, the advanced technique of revascularization together with new drugs can significantly reduce the risk. Pilots must also undertake aggressive healthy lifestyle modifications. The 4 × 4 aeromedical risk matrix is a very useful tool that can aid in decision making for the assessment and recertification process. The initial recertification process can be completed after 3 months of the revascularization procedure; however, it is limited to pilots who have no MI, uncomplicated MI or undergo PCI that excludes the left main coronary artery. Otherwise, it is strongly recommended to wait for at least six months after revascularization before assessing pilots for return to work. Functional imaging is an essential investigation prior to the recertification process to evaluate the residual disease after revascularization. A flying restriction to dual pilot operations is possible if the result of functional imaging is acceptable.

## CONFLICT OF INTEREST

The authors have declared no conflicts of interest relevant to this article.

## AUTHOR CONTRIBUTIONS

ZM and RI conceived and designed the study. ZM drafted the manuscript. MRMR and MHH critically revised the manuscript. RI supervised the study.

## DISCLOSURES

*Ethical approval*: N/A. *Informed consent*: All participants provided written informed consent for their information to be included in the study. Confidentiality was maintained throughout the study by not recording participant names. *Registry and the Registration No. of the study/Trial*: N/A. *Animal Studies*: N/A.
